# Analecta

**Published:** 1842-09-10

**Authors:** 


### ANALECTA.

*Treatment of Facial Hemiplegia, by Division of the Muscles on the opposite side of the face.* By M. Dieffenbach.—M. Dieffenbach having observed that facial hemiplegia, when of long standing, or depending on an alteration in the structure of the portia dura nerve, often resists every method of treatment that has been hitherto proposed, had long conceived the idea of remedying by operation the deformity which results from it. His first experiments with this view were made on two patients, one an old, the other a young man, in both of whom this affection produced considerable distortion. He excised an elliptical portion from the paralysed cheek, and united the edges by suture; the wound healed in a few days, with a decided improvement in the physiognomy of the two individuals. The affected cheek, shortened by the loss of its substance, was enabled in some measure to antagonize the muscles of the opposite side, though the actions of speaking, eating, and laughing, were sufficient to destroy the equilibrium. This operation was too exclusively mechanical in its nature, and insufficient in its effect, long to satisfy the surgeon. Recent operations had given M. Dieffenbach occasion to observe, that by the loss of their natural antagonists, healthy muscles are wont to become more firm and contracted: this led him to draw an analogy between the consequences of the present affection and the contraction of muscles in certain cases of club-foot, which takes place at the expense of their paralysed opponents. This analogy suggested the idea of a similar operation, and success soon justified his anticipation. The attempt was first put in practice on a man who had long been the subject of facial hemiplegia, which was at that time confined to the upper eyelid. This part remained permanently raised, the man being unable to close the eye; the orbicularis muscle, which is dependent on the facial nerve, had lost its power, while the levator palpebræ, supplied from a different source, retained its action. M. Dieffenbach accordingly determined to divide the latter muscle; this was readily effected by a small incision on the outer side of the eyelid, just above the tarsal cartilage, in such a manner that the fibres of the levator were divided transversely, while those of the orbicularis were only interfered with in the line of their direction, and consequently but little injured. The result of the operation was surprising; the man could immediately open and close the eyelids: this sudden effect showing that the orbicularis had not been completely paralysed, and that some fibres of the levator remained undivided. In three other operations the success was equally striking, and the benefit permanent. Scarcely any blood was lost by the operation, and the cure was complete in three or four days. In the fourth case the incision was followed by erysipelatous inflammation and suppuration, and the subject of it was only imperfectly cured after several months.

Encouraged by these results, M. Dieffenbach did not confine his operation to the levator palpebræ in cases of hemiplegia, but applied the same to other muscles retracted in consequence of this affection. The same mode was adopted as had been before employed by him in cases of convulsions of the muscles of the face, namely, subcutaneous incision. A small opening was made through the integuments of the middle of the cheek, in a line between the angle of the mouth and the lobe of the ear; through this a thin knife was introduced, and carried beneath the skin as far as the external canthus, and in withdrawing it all the muscles in this situation were divided. By another incision made at the angle of the mouth, the muscles at the lower part of the face were cut across in an oblique line from above downwards, to the lower edge of the inferior maxillary bone. One of the remarkable results of this operation was, that the paralysed muscles, previously lax and inactive, immediately regained their energy in some degree, and were even able to execute some movements. The wounds were covered with charpie, retained in position by adhesive plaster; union taking place in a few days. The success generally surpassed the expectations of the operator; it was least remarkable in cases where the paralysis had been of long standing. The least fortunate attempt was made on a woman, 34 years of age, who suffered under facial hemiplegia almost from birth. The deformity was extreme, the face being much drawn to the right side, and the eye kept widely open day and night. M. Dieffenbach divided the corresponding levator palpebræ, and the contracted muscles of the sound side of the face. The immediate benefit of the operation was very striking; the movements of the eyelid were restored, as well as those of the paralysed cheek, sufficiently to allow the patient to laugh without its occasioning distortion of her features. But the success was not permanent; the deformity of the face soon returned, and a second operative attempt was made with no better result as regarded the appearance of the face; but the eyelid retained its mobility, and this was considered by the patient a sufficient recompense for the suffering she had undergone.—*Lond. Med. Gaz., from Medizinishe Zeitung.*

---

*Properties of Ergot of Rye.*—M. Boujean, pharmaceutist at Chambery, presented to the Academy of Sciences a memoir on the toxicological and medical history of the ergot of rye, in which he classes this substance among narcotics, and says that its effects bear close relation with those of morphia, though it contains no trace of that alkaloid.

The most interesting result from the researches of M. Boujean is, that the ergot of rye contains two active principles, distinct from one another—a remedial and a poisonous agent. The first is a reddish brown extract, very soluble in hot water, which possesses in the highest degree the valuable obstetrical and hemostatic properties, which have long been recognized in the ergot. The other is a fixed colourless oil, very soluble in cold ether, insoluble in boiling alcohol, in which alone reside the poisonous properties of this substance. The different nature of these two products admits of their easy separation, and of the remedy being obtained entirely isolated from the poison. As the former is altogether inoffensive, great advantage thus results to practical medicine, that a large dose may be administered without the fear of any of the accidents which are attributed to the ergot of rye itself. This extract acts with extreme rapidity in all hæmorrhages, without producing any unfavorable action, whatever may be the quantity employed. M. Boujean has repeatedly administered large doses in severe hæmorrhages, followed by abortions or otherwise, which instantly yielded to the influence of this remedy. He therefore designates it the *Extrait hémostatique.*

The oil acted on animals in the same manner as the ergot itself, only its effects were more prompt. They were almost immediate in some animals, as birds. They died in about twenty-four hours, without ever recovering from the state of stupor into which the poison had plunged them. To obtain this oil with all its properties, it is necessary to extract it with cold ether, and in the operation to avoid all influence of heat; and the principle will be found altogether inert if obtained from ergot which has not arrived at maturity.—*Ibid., from Gazette Médicale.*

---

*Treatment of Umbilical Hernia in Children by Ligature.*—A child, aged 8 months, was brought to M. Bouchacourt suffering under umbilical hernia, which had been observed a few days after birth: various means had been employed to keep the swelling reduced and to effect a cure, without producing any benefit. The hernia easily protruded, and formed a considerable swelling, When it was returned, the finger readily entered the unobliterated ring, and felt its smooth and regular edge. The operation was conducted as follows:—The child being secured and the hernia returned, the surgeon assured himself by careful examination that no intestine or other viscus remained in the sac, by rubbing its sides against one another between the finger and thumb. Keeping up a pressure with the finger close by the ring to prevent the protrusion of any part into the sac, a needle armed with double thread, was passed through the base of the projection in the integuments into which the hernia protruded as into the finger of a glove, and the threads being separated, each was tied upon the corresponding half of the swelling. The base was also enveloped by a third thread carried round the whole, and drawn tight. The child did not appear to suffer much—only a small piece of lint placed on the part. The first night the infant cried and slept little, but afterwards went on well, with the exception of slight fever in the evening, and a diminution of appetite, no alteration being observed in its other functions. The stools were regular, and it had no vomiting. After a few days the encircled part swelled, sloughed off, leaving a considerable ulcer. In two months and two days after the operation, a very small surface remained unhealthy, giving vent to a discharge which scarcely tinged the linen. The ring appeared to be obliterated. The hernia had not returned, and from the day on which the ligature was applied, the swelling had not once shown itself, notwithstanding the efforts and cries of the child.—*Ibid, from Reveu Médicale.*

---

*Employment of Ergot of Rye in a Case of Hydatid Mole.*—Madame H., eighteen months after marriage, suffered under a suppression of the menses. She had vomiting, and a distaste for food. Two months afterwards, the abdomen having swelled to a considerable size, the suspicion of pregnancy was thought to be confirmed. At the end of four months she experienced acute pain in the loins, and uterine hæmorrhage commenced. M. Cabaret was called in. On his arrival the discharge of blood had ceased, but the pain continued. From the unusual rapidity with which the increase in the size of the abdomen had taken place, and the presence of a soft substance which could be distinguished within the hard and undilated neck of the uterus, he judged the presence of a mole. Shortly afterwards the evacuation of certain portions of cysts confirmed his opinion; and having unsuccessfully employed several means to excite uterine contractions, he had recourse to the ergot of rye, which, in a few hours, effected the expulsion of the entire mass. It consisted of a number of hydatids, or vesicles with thin transparent walls, easily broken down, and filled with a limpid, slightly albuminous fluid.—*Ibid., from Revue Médicale.*

---

*Additional Observatious on Fibre.* By Martin Barry, M. D. On examining coagulating blood, the author finds that it contains discs of two different kinds; the one comparatively pale; the other very red. It is in the latter discs that a filament is formed; and it is these discs which enter into the formation of the clot; the former, or the pale discs, being merely entangled in the clot, or else remaining in the serum. He thinks that the filament escaped the notice of former observers, from their having directed their attention almost exclusively to the undeveloped discs which remained in the serum, and thus conceived that the blood-discs are of subordinate importance, and are not concerned in the evolution of fibrin.

To render the filament distinctly visible, Dr. Barry adds a chemical reagent capable of removing a portion of the red coloring matter, without altogether dissolving the filament. He employs for this purpose chiefly a solution of one part of nitrate of silver in 120 parts of distilled water; and sometimes also the chromic acid. He admits that the use of these re-agents would, on account of their destructive tendency when concentrated, be objectionable as proofs of the absence of any visible structure; but as the point to be proved is, that a certain specific structure does exist, he contends that the same appearance would not equally result from the chemical actions of re-agents so different as are those of chrome and the salts of mercury and of silver. After the appearance of the filament, thus brought to light, has become familiar to the eye, it may be discerned in the blood-discs when coagulation has commenced, without any addition whatever. Those blood-discs of the newt, which contain filaments, often assume the form of flask-like vesicles, the membranes of which exhibit folds, converging towards the neck, where, on careful examination, a minute body may be seen protruding. This body is the extremity of the filament in question, its protrusion being occasionally such as admit of its remarkable structure being recognised.

The author proceeds to describe various appearances which he has observed in the coagulum of the blood, and which strongly resemble those met with in the tissues of the body, and are obviously referable to a similar process of formation. He bears testimony to the accuracy of the delineations of coagulated blood given by Mr. Gulliver. One of the most remarkable phenomena discovered by the author in the coagulation of the blood is, the evolution of red coloring matter—a change corresponding to that which he had previously observed to take place in the formation of the various structures of the body out of the corpuscles of the blood. He considers the production of filaments as constituting the essential circumstance in coagulation.

He conjectures that the notched or granulated fibres noticed in the blood by Professor Mayer, may have been of the same kind as the flat, grooved, and compound filaments described by himself; but he thinks that in that case, Mayer's explanation of their mode of origin must be erroneous; for they may be seen to be produced by a portion of the blood not mentioned by him—namely, the corpuscles.

Mr. Addison's discovery of globules in the uppermost stratum of inflammatory blood, and of their influence in the formation of the buffy coat, is confirmed by Dr. Barry, who remarks that these globules are altered red blood-discs. That the blood corpuscles are reproduced by means of parent-cells, as suggested by Mr. Owen and by the author, is confirmed by the observations of Dr. Remak; but the author had long ago indicated a division of the nucleus as being more particularly the mode of reproduction, not only of those corpuscles, but of cells in general. With this conjecture the observations of Remak on the blood-corpuscles of the fœtal chick fully accord. Whether the author's further speculation—namely, that the parent-cells are altered red blood-discs, is correct, still remains to be seen.

The phenomenon of the "breaking off short," or notching of the fasciculus of a voluntary muscle in a transverse cleavage of the fibre, is regarded by Dr. Barry as a natural consequence of the interlacing of the larger spirals, which he has described in a former paper; the fracture, in proceeding directly across the fasciculus, taking the direction in which there is least resistance.

The position of the filament in the blood-corpuscle is represented as bearing a striking resemblance to that of the young in the ovum of certain intestinal worms, the filaments of which are reproduced by spontaneous division.

The author subjoins the following quere: "Is the blood-corpuscle to be regarded as an ovum?"—*Prov. Med. Jour.*, July 9, 1842.

---

*Strabismus.*—At a branch meeting of the Provincial Association, held at Bath, Mr. Estlin read a communication on strabismus, giving the result of his experience in 100 cases in which he had operated. Of these, 39 were males, and 61 females; and their ages were as follows:—From five years and a half to ten, 13; ten to fifteen, 17; fifteen to twenty, 23; twenty to thirty, 26; thirty to forty, 9; forty to fifty, 5; fifty to sixty, 5; sixty-two, 1. In these cases, 39 squinted in the left eye; 22 in the right; and the rest in both eyes. The results of these operations had been—internal squint perfectly cured, 44; satisfactory, 21; very satisfactory, 9; improved, but requiring another operation, 7; squint returned, 4; improved, but unfavorable for a second operation, 5. Of external squint, 5 had been perfectly cured, 3 improved, and 1 not at all improved. On the whole number of cases, then, the result was—perfectly cured, 65; satisfactory, 9; improved, but requiring another operation, 7; not improved, requiring operations on both eyes, 4; unfit for operation, 5; no improvement, 2; slight improvement, 3. The operations for external strabismus had not been so successful as those for internal; but then the proportion of cases was fortunately very small. The paper concluded by some well-timed remarks on the moral influence achieved by this triumph of surgery.—*Ibid.*

---

*Rupture of the Uterus, caused by the improper use of the Secale Cornutum.*—M. Delmas has published, in the "Journal of Practical Medicine, of Montpellier," two cases of ruptured uterus, caused by the unadvised administration of the secale cornutum. One of these will serve as an example or beacon against hurrying or interfering with labor when all is doing well. A young woman, twenty years of age, primiparous, having always enjoyed good health, and having gone her full time, was taken in labor under the most favorable circumstances. After the lapse of eight hours, the os uteri was nearly quite dilated, the bag of waters had broken, everything was proceeding as it should, when her attendant decided to give a scruple of powdered secale to hasten the egress of the child. The uterine contractions were increased, but the head receded into the pelvic cavity, and was replaced by a soft, unequal tumor. M. Delmas having been sent for, found the poor woman in a state of extreme anxiety, complaining constantly of general malaise, with sickness and fainting; the pulse was small and contracted, the abdomen painful to the touch, and containing two distinct tumors, one to the right, the other to the left; the smaller one to the left was the nearer to the surface, and the more readily circumscribed; the larger one to the right was less hard, deeper seated, and presented a less regular surface. A small quantity of blood drained away from the vagina, and on examination it was found that, although the os uteri was dilated, the right shoulder presented only at the brim of the pelvis. M. Delmas at once conjectured that rupture of the uterus had taken place, and having roused the vital powers by a cordial, brought down the feet, and delivered his patient of a dead child. While thus proceeding, M. Delmas, having one hand on the tumor on the left side, felt it re-enter the uterine cavity. The poor woman died a few days afterwards.—*Ibid.*

*Corpora Lutea.*—Dr. Montgomery, after the most extensive experiments and elaborate investigations, states that conception never happens without the production of a corpus luteum, and that the corpus luteum is never found in virgin animals, but is the effect of impregnation. This opinion Mr. Renaud seeks to controvert. He admits that impregnation never does occur without our being able to detect one of these bodies, but he is opposed to the concluding part of Dr. Montgomery's statement, that the corpus luteum is never found in virgin animals. In support of his own opinion, he narrates the appearances after death in a young sheep, which he judges to have been a virgin from internal evidence. A true corpus luteum was found in the left ovary, the right containing a spurious one. The uterus was remarkably small, and on being opened, proved to be destitute of any traces of an embryo, nor was its cavity lined with the ropy mucus, but simply moistened, as is the case with all visceral cavities lined with mucous membrane. The cavity of the uterus was completely covered with a deep black lining, except here and there on the summits of a number of round or oval elevations, which are the maternal cotyledons, into which the fœtal tufts penetrate during pregnancy. The black coating is an epithelial structure, consisting of two distinct varieties of cells arranged in a peculiar manner. The dark matter is entirely made up of pigmentary globules, which together form a kind of net-work, in the interspaces of which the simple nucleated cells of Schwann are seen in great numbers ; Mr. Renaud considers the precise appearance of this epithelium of consequence, as much of the correct diagnosis depends on it. It exists in the fully developed unimpregnated uterus of the ewe, although it is not found in earlier life, but he is not aware whether it appears subsequent to pregnancy.

Two objections he thinks may be alleged against his case, the first that an early ovum might have been contained in the uterus, and overlooked or lost from its small size ; the other is, that abortion might have taken place. To the first, Mr. Renaud alleges the care taken in the examination, the size of the uterus, and the presence of the epithelial lining—beside which, the size and perfect character of the corpus luteum would require a fœtus a month old. The second objection he combats by the state of the uterus.

He adds that the following appearances ought to be clearly discernible previous to any professional evidence being given in a case, whether it be moral, forensic, or physiological :—First, a distinct external envelope, in contact, and in union with the stroma of the ovary, but capable of being dissected away from it entire. Secondly, a solid substance, either fleshy-looking, red, pinkish, or yellow-colored, which should be divided into a greater or less number of segments or lobuli. The deeply fissured appearance of these lobuli has been insisted upon, but as in a great many instances it is absent, its presence can afford no positive criterion. Thirdly, an inner membrane, or the proper ovisac thickened. Fourthly, a central deposition of granular or other matter, or the remains of it. Fifthly, the microscopic appearance presented will form a very good auxiliary. The fact of one of the radii reaching as far as the surface of the ovary, is an appearance useful as an auxiliary diagnostic indication, but it is equally present in the false as in the true bodies.—*London and Edinburgh Monthly Medical Journal.*

---

*Spontaneous Obliteration of the Axillary Artery.* By Dr. Oke.—A lady aborted on the 7th November, 1831. On the afternoon of the 8th, she felt a sensation at the extremities of the fingers, as if they were scorched. The integuments at the points felt hard, looked white, and were painful, so that she apprehended she was going to have whitloe in all her fingers. She awoke on the following morning from a disturbed sleep, with intense pain and numbness of the whole arm, and almost total blindness. The left arm was now cold and insensible. The wrist and the tops of the fingers were growing discoloured: the ring-finger especially was becoming black. And now no pulsation could be felt in the axillary artery, or any of its branches, below the superior margin of the pectoralis major. This was on the 9th. On the 13th, the sense of feeling and temperature began slowly to return, though the arm still continued very painful; and the discoloration declined: but the ring-finger became black and hard at its extremity, and around the dry crust at that point appeared a line of separation, consisting of a slightly elevated circle, containing a minute quantity of pus. The top of this finger exfoliated, like a dry, hard, black, horn button, nine months after the obliteration of the vessel. So did the integumental extremities of the thumb, first, second, and last fingers. The author thinks the obliteration of the artery is to be accounted for from sudden spontaneous rupture of the internal coat of the artery.—*British and Foreign Medical Review.* July, 1842.

---

*Dislocation of the Hip into the Ischiatic Notch in a Child.* By Clement J. Hawkins.—A. B., aged seven and a half years, was admitted into St. Bartholomew's Hospital, April 30th, 1836, with an injury of the hip, which had taken place about an hour previously.

On examination, the left leg was found to be about an inch and a half shorter than the right, the knee bent across the opposite limb, the foot a very little turned inwards; when the boy was made to stand up, the leg remained much in the same state, the toes barely reaching the ground; the head of the femur can be felt resting near the great ischiatic notch; there is considerable power of flexion remaining, but the limb cannot be rotated outwards to any extent.

The accident was occasioned in the following way:—He was carrying another lad on his back, his foot slipping into a hole he fell forwards with his burthen.

The case was considered by all present to be one of dislocation of the head of the femur into the ischiatic notch.

The reduction was attempted by the house surgeon and dressers, but failed, owing to the difficulty of fixing the pelvis.

Mr. Skey was sent for, who dexterously reduced the luxation in the following manner:—The patient was placed on a high table lying on his side; a jack-towel was placed between the scrotum and thigh (being twisted;) it was firmly held by an assistant; a napkin was placed beneath this, and held by the house surgeon in such a direction as to prevent the bone pressing in the groin; extension was steadily kept up, from the ankle, by two assistants, Mr. Skey gently elevating and rotating the limb outwards. By these means the head of the bone quickly returned to its socket.

*Remarks.*

This case occurred during the year I was a dresser to the late Mr. Henry Earle, at St. Bartholomew's Hospital. In a clinical lecture delivered at the time of the accident, Mr. Earle observed he had never himself met with a similar accident, but mentioned one which happened in the practice of an eminent provincial surgeon (Mr. Norman, of Bath, I think,) the subject being only four years of age.

Some years since, through the kindness of my friend, Mr. Eves, of this town, I witnessed a well-marked instance of dislocation of the femur into the ischiatic notch, in a youth whose age I do not recollect, but I firmly believe he was not more than ten or eleven years of age.

In the case I have related some discussion took place as to the situation of the dislocated bone. Mr. Skey would not undertake to say that the head of the bone rested in the notch, but was rather inclined to think it rested close on its border; however, I think it corresponded well with the description given by Sir A. Cooper of the symptoms of dislocation into the ischiatic notch.—*Provincial Medical Journal.* July 30, 1842.

---

*Superficial Cancers.*—M. Lisfranc has published a series of cases of cancer, in which under ordinary circumstances it would have been necessary to remove the diseased organs, but which he has shown, admit of permanent cure by the simple ablation of the part of the organ which is diseased. The first case he mentions is that of a man who consulted him in 1826, having a cancerous tumour, about half an inch thick, surrounding the penis behind the gland; its antero-posterior diameter was about two inches. It was ulcerated, immovable, and adherent, and presented all the characteristics of cancer. As the removal of the penis is an operation to which the greatest repugnance is always manifested, Lisfranc decided on making an exploratory incision, and by a careful and slow dissection lay bare the corpora cavernosa; if they were sound he would then proceed to extirpate the diseased growth, if not, he would amputate the penis. The additional pain of this proceeding would be well compensated by the prospect of retaining so important an organ. The operation succeeded remarkably well; the cavernous structure was healthy, only a small portion of the fibrous covering of the penis, where ulceration had taken place, being engaged in the disease, which was removed. The patient had not a bad symptom afterwards, and recovered perfectly in the course of three weeks. He showed himself to Lisfranc several times afterwards, and assured him he was perfectly competent to perform all his duties.

The successful termination of this case encouraged the surgeon to proceed in like manner with a patient who laboured under a cancer of greater extent. It occupied the anterior portion of the scrotum, and about two inches of the skin of the root of the penis, and the posterior half of that organ. Every part was ulcerated, and the disease was of long standing. By a careful, slow, and laborious dissection, the testicles and spermatic cords were uncovered; the cancerous portion of the scrotum being cut away, the next step was to make a similar exploration of the penis, which being done, showed that the superior ligament was diseased, and must be removed. The carcinoma extended as far as the reunion of the corpora cavernosa, which were necessarily denuded as for an anatomical lecture, and even then it was requisite to scrape them with a bistoury to remove all traces of the disease. The patient recovered in rather less than seven weeks, preserving the virile power.

Cancer of the tongue has hitherto been treated by the ablation of the organ; but Lisfranc has demonstrated that, in some cases at least, the disease is superficial, and may be removed without permanent injury to the organ. He narrates the case of a young advocate, who was under his care in September, 1826, for an ulcerated cancer occupying the two-thirds of the right side of the tongue. Its ablation had been advised by several eminent surgeons, but Lisfranc decided otherwise. He separated the diseased from the healthy structure, and surrounded the former with a ligature, which was tightened by Mayor's tourniquet. The constriction was gradually increased, and on the seventh day, the slough having separated, there was a loss of only two lines in extent of the point of the tongue. The surface only was diseased; the deeper tissues were healthy, and cicatrised. This patient was seen by Lisfranc three years after the operation, and no relapse had occurred.

Several other cases are recorded by this talented surgeon, including cancer of the penis and vagina, of the ala nasi, the eyelids, the loins, the finger, &c., in all of which the primary exploratory incision showed clearly that the disease was superficial, and admitted of its total ablation, without the absolute destruction of the organ itself. At the same time Lisfranc states that while some of these cancers are regarded as deep-seated erroneously, the latter condition may exist, and require the complete amputation of the part.—*Ibid, from Clinique Chirurgicale.*

---

*On Spontaneous Fracture of the Thigh-bone.* By JONATHAN TOOGOOD, Esq.—In the course of my practice, two cases have occurred in which the thigh has been fractured without any external violence. The first was that of a man who had for many years been in a weak, nervous, and half paralytic state. In attempting to turn in bed, the bone broke. The case was considered an extraordinary one; it was treated in the usual way, and united after a considerable time, and he lived many years after. The next case was that of James Pople's wife, of Bawdrip, aged fifty-five, who had been long in an infirm state of health, which terminated ultimately in paralysis of the lower extremities. She had suffered very severe pain in her right thigh for some months, which was considered by those about her to be rheumatic; and being a poor woman without friends, little was done for her relief. One evening, on being lifted up in bed, the bone suddenly snapped; she was aware of it immediately, and cried out that her thigh was broken, but no one believed her, and she lay all that night in dreadful agony, but when, on the following morning, her neighbours saw the limb almost doubled by the violent spasmodic action of the muscles which drew the ends of the bones forcibly against each other, I was requested to see her. Her condition was indeed truly deplorable, and the grating of the bones against each other was distinctly heard.

The limb was placed in splints, and united after a longer period than usual. I mentioned this case to Sir Astley Cooper, who considered it to be cancer of the bone, and directed my attention to the state of the breasts, in both of which I discovered on examination several hard, knotty tumors, of a carcinomatous character.

The following interesting account of a similar accident will be found in the life of Archbishop Secker:—

About a year and a half before he died, after a fit of the gout, he was attacked with a pain in the arm, near the shoulder, which having continued about a twelvemonth, a similar pain seized the upper and outer part of the opposite thigh, and the arm soon became easier. This was much more grievous than the former, as it quickly disabled him from walking, and kept him in almost continual torment, except when he was in a reclined position. During this time he had two or three fits of the gout, but neither the gout nor medicines alleviated these pains, which, with the want of exercise, brought him into a general bad habit of body.

On Saturday, the 30th of July, 1768, he was seized, as he sat at dinner, with a sickness at his stomach. He recovered himself before night, but the next evening, whilst his physicians were attending, and his servants raising him on his couch, he suddenly cried out that his thigh-bone was broken. The shock was so violent, that the servants perceived the couch to shake under him, and the pain so acute and unexpected, that it overcame the firmness he so remarkably possessed. He lay for some time in great agonies, but when the surgeons arrived, and discovered with certainty that the bone was broken, he was perfectly resigned, and never afterwards asked a question about the event. A fever soon ensued ; on Tuesday he became lethargic, and continued so till about five o'clock on Wednesday afternoon, when he expired with great calmness in the 75th year of his age.

On examination, the thigh-bone was found to be carious about four inches in length, and at nearly the same distance from its head. The disease took its rise from the internal part of the bone, and had so entirely destroyed its substance, that nothing remained at the part where it was broken but a portion of its outward integument ; and even this had many perforations, one of which was large enough to admit two fingers, and was filled up with a fungous substance, arising from within the bone. There was no appearance of matter about the caries, and the surrounding parts were in a sound state. It was apparent that the torture which his grace underwent during the gradual corrosion of this bone, must have been inexpressibly great. Out of tenderness to his family, he seldom made any complaint to them, but to his physicians he frequently declared his pains were so excrutiating, that unless some relief could be procured he thought it would be impossible for human nature to support them long ; yet he bore them for upwards of six months with astonishing patience and fortitude, sat up generally the greater part of the day, admitted his particular friends to see him, mixed with his family at the usual hours, sometimes with his usual cheerfulness, and, except some very slight defects of memory, retained all his faculties and senses in their full vigour till within a few days of his death."

In the second part of the 15th vol. of the Medico-Chirurgical Transactions, two cases of fracture of the thigh-bone taking place, without any violence, in connection with cancer, are related by Mr. Salter, of Poole, in one of which an examination was afforded after death, and his description of the condition of the bone corresponds very much with that of the preceding case. I regret that no examination could be obtained in the case of James Pople's wife.—*Prov. Med. Jour.*, July 9, 1842.

---

*Recovery of motion after Resection of the Elbow-joint.*—At the Academy of Medicine, Paris, July 5, M. Robert, surgeon to the hospital Beaujon, presented a female, aged twenty-six, on whom he had performed the operation of resection of the elbow-joint.

After a fall on the elbow, the joint had become carious, and two fistulous openings had formed, but the surrounding tissues were not much tumefied.

The method adopted was very similar to that described by M. Velpeau, in his work on operative medicine. The humerus was sawn through immediately above the condyles ; the ulna below the coronoid process, and the radius a little below its head, so that about four inches of the bone were removed. The limb was now placed in M. Guyot's apparatus ; no reaction took place, and even the febrile symptoms, which were present before the operation, disappeared.

The discharge of pus continued for a considerable length of time, and as it was impossible to bring the bones together, the wound did not heal before the expiration of eighteen months. Two years and a quarter have now elapsed since the operation; and it is a matter of some interest to observe the manner in which the motions of the forearm and hand are executed. The limb is now nearly of the same size as the opposite one; when the arm is allowed to hang down by the side, a separation of about three inches may be felt between the extremity of the humerus and the upper part of the bones of the forearm. In this position the arm looks, as it were, paralysed ; but when the patient makes an attempt to bend it, the space between the divided bones seems to disappear in consequence of the ascension of the forearm ; the ends of the radius and ulna obtain a point of support on the extremity of the humerus, and flexion takes place to the extent of a right angle. The patient carries the hand easily to the head, or to the shoulder of the opposite side, and can raise a heavy weight with it. The movement of pronation is pretty free, and those of the fingers perfectly so. Within the last few months the patient has been able to resume her occupation of semptress.—*Ibid.*

---

*Nervous Aphonia.*—Aphonia, dependant on a disordered condition of the nerves of the muscles connected with the function of speech, comes on suddenly, and immediately after the influence of the exciting cause has been exerted. In the majority of cases the loss of voice is complete and absolute; the patients are either entirely mute, or can only produce very feeble sounds. There is not any pain in the larynx, any difficulty of breathing, cough, or morbid secretion. It is very frequently intermittent, coming on suddenly and disappearing in the same manner, often under the influence of a lively moral impression. There are several varieties of nervous aphonia. The idiopathic, dependant on lesion of function of the laryngeal nerves and muscles, is divided by M. Hirtz into hypersthenic, and hyposthenic. The first is induced by exciting causes, and shows itself in irritable and sanguine temperaments. It comes on suddenly, is commonly attended with a feeling of strangulation, and has little tendency to a chronic state. The other variety of idiopathic aphonia—the hyposthenic or paralytic form—is brought on by debilitating causes, is more obstinate, and more difficult of removal. Stimulating frictions over the larynx, the inhalation of steam, blisters, frictions with croton oil, aluminous and benzoic insufflations are recommended, and should be tried in the first instance before having recourse to more powerful measures. On their failure, setons, electro and galvano-puncture, strychnia and the substances containing it, may be employed.—*Ibid, from Journal de Méd.*

*Erysipelas.*—Considered in a surgical point of view, erysipelas depends more as regards its predisposing cause on external, atmospheric, or meteorological inflammations, than on the state of the general health or constitution of the patient. Its determining or occasional cause is almost always a wound or irritation of some part of the skin, the efficient cause being generally some matter coming from without or from disorganised tissues, and mixing either primarily or secondarily with the fluids of the diseased part. The fluids thus changed produce two orders of morbid phenomena, those indicative of general disorder, and local disease. An isolated spot of erysipelas is generally removed in from four to six or eight days, but as the entire inflammation is composed of a number of successive erysipelatous spots, its duration varies in proportion to their number.

With regard to the treatment, Velpeau has kept notes of 400 cases treated by different plans. Of these, 25 were subjected to compression; 33 to the successive application of blisters, either upon the immediate seat of the inflammation, or around it; 30 were treated by the nitrate of silver, either in substance or solution, sometimes applied on the surface, sometimes around it. In two cases the actural cautery was used, and in all these the remedies were without satisfactory results. The Neapolitan ointment was tried in 200 cases, and was found to shorten the duration of the complaint a day or two, and perhaps to render it rather less painful; but its application is repugnant to the patient, gives rise to salivation, and spoils the linen. Twenty-three cases were treated with purified fresh lard, which was less efficacious than the mercurial ointment; 12 by the white precipitate ointment, which increased the disease; 10 by very diluted sulphuric acid; 6 by citric acid, tartaric acid, oxicrat, and salt water; 6 by the acid nitrate of mercury, used as a lotion in 3 cases, and in the others as a slight caustic around the circumference of the inflammation, but all unavailingly. The application of camphor, and puncturing the part were equally useless.

Velpeau, from the experience gained in the treatment of 40 cases, recommends the sulphate of iron, which, he says, has really arrested the progress of the disease in the majority of cases. He employs it in solution in the dose of thirty scruples to a quart of water, and in ointment, containing eight scruples of the chalybeate to thirty of lard. The ointment is not so useful an application as the solution, bnt it is more convenient when large surfaces on the body, neck, or head, require its use. The entire surface of the inflamed part and a little beyond it, should be anointed with it three times a-day. The solution is employed in lotions, compresses wetted with it, being applied every six hours, so as to keep the part constantly moistened.—*Ibid, from Annales de Chirurgie.*

---

*Spinal Irritation in Intermittent Fevers.*—Dr. Gouzée has published an important communication on the morbid sensibility of certain portions of the spine in cases of chronic intermittents in the " Annales de la Société de Médicine d'Anvers," in which he states that this remarkable and little known fact of the production of pain by pressure on a particular point of the spinal column in agues has a direct reference to practice. The manipulation, according to him, requires considerable attention and practice; each of the spinous processes must be examined successively from above downwards, the apices of the index and middle fingers being pressed perpendicularly on the top of the process in its recognised direction, the compression being applied gradually and equally on all. In general the patient is not aware of this morbid sensibility, and bears the examination of the cervical vertebræ with indifference, but as soon as the operator commences with the dorsal, he shrinks and gives evident indications of pain. Most frequently, while two or more vertebræ are tender to the touch, one of these will evince a higher degree of irritability than the rest, as may be ascertained by repeated examinations. The same amount of pressure, exercised on the lowest range of vertebæ, will not cause any appearance of suffering.

In illustration of these statements, Dr. Gouzée narrates several cases in which antiphlogistics and counter-irritants, applied near the painful portion of the spine, produced the most beneficial results. Leeching, cupping, and the Burgundy pitch-plaster sprinkled with tartarised antimony, so as to produce pustulation, where the remedial agents had recourse to, and were found of exceeding service. In the cases recorded, each application of leeches was followed by a marked relief of the symptoms, a diminished intensity of the febrile accessions. In one case, pressure showed the existence of morbid irritability of the spine in the neck, as well as in the dorsal vertebæ.—*Prov. Med. Jour.* June 25, 1842.

---

*On Cysts occurring in the Neck, but not necessarily connected with the Thyroid Body.* By B. Phillips, F. R. S.—The object of this paper is to show that there are encysted tumors developed in the neck, commonly after the prime of life, and having no necessary connection with the thyroid body, although in their progress they may implicate that organ to a considerable extent. Those cysts are usually first seen at a certain distance from that organ; and in obtaining a history of the disease at a time when it presents the appearance of a thyroid tumor, care must be taken to ascertain this point. The cyst is filled with a serous fluid, varying in color from straw to a dark, coffee-like appearance, and coagulable by heat. It may acquire a very large size; in one of the cases which occurred to the author of the paper, it was estimated that the contents amounted to six or seven pints; but even then it is the bulk only which interferes with neighbouring organs. The fluid may be discharged by puncture, but the tumor will refill; injections are either too stimulating, and produce much disturbance, or are too unirritating to modify the surface of the cyst. The plan first used by Mr. Hill, of Dumfries, of passing a thread or two through the cyst, so as to form a seton, seems to be the best mode of treatment. The paper details several cases and some dissections.

Mr. Dalrymple observed, that the puncture of the cysts described by Mr. Phillips, was not always unattended by danger, as the result of a case which occurred a few years ago, in his father's practice, would illustrate. A man, healthy and of the middle age, presented himself to the notice of Mr. Dalrymple, of Norwich, on account of a large fluctuating tumor of the neck. It reached from the lobe of the right ear to the collar-bone; was irregular in shape, and at the lower part overlapped the clavicle, and presented at this point a round, well-defined tumor, as large as a Seville orange. It had already, by its bulk, produced great dyspnœa, and had pushed the larynx and trachea over to the left side. The tumor fluctuated at all points, but had no pulsation. It was thought advisable to puncture the swelling, and a large quantity of straw-colored serum was evacuated by a small opening at the most dependent point. Its size was immediately reduced, and the breathing became free: the incision was closed, and the patient was on the point of going away, when it was observed to have attained nearly its original bulk, and the wound being again opened, a large gush of florid, arterial blood ensued. The hæmorrhage was with difficulty restrained; and the late Mr. Martineau being called into consultation, pronounced the tumor to have been an aneurism of the carotid artery. A compress, however, arrested the bleeding, and Mr. Dalrymple not agreeing in that opinion, the patient was sent to bed, and constantly watched. In two days hæmorrhage again took place, and which was restrained as before, by renewal of the dressings. Great constitutional irritation ensued, and, although no considerable loss of blood took place after the second bleeding, the patient gradually sunk, and died in a typhoid condition at the end of about sixteen days. He (the speaker) examined the disease himself, and found it to consist of a multilocular tumor, originating in the right lobe of the thyroid gland. This organ seemed to have been converted into numerous large and irregularly-shaped cysts, which extended from the lobe of the ear, which it displaced upwards, to the clavicle, which it overlapped below. It was continuous with the isthmus of the gland; but no trace of normal structure belonging to the right lobe existed. The left lobe was slightly enlarged, and a few watery cysts, not larger than a pea, were found in its otherwise healthy structure. The great vessels of the neck were entirely unconnected with the tumor, and colored fluid injected into the arteria innominata did not pass into the cysts. On opening the cysts, however, a large, elongated fungoid mass presented, hanging by a pedicle about an inch and a half long, attached to the walls of, and depending into the cavity of one of the larger cysts, and from this polype the bleeding probably took place. It was conjectured, and he thought with truth, that the loose and tender vessels of this fungoid mass, losing the support of the previously contained fluid, gave way after the evacuation of the serum, and originated the serious hæmorrhage which led to the fatal result. The case showed, first, that these cysts were sometimes connected with, or arose in the substance of the thyroid body; and, secondly, that the evacuation of them was not unattended with danger. It also forcibly pointed out the difficulty of diagnosis in anomalous tumors of the neck, where arterial hæmorrhage followed the puncture of a cyst, since so eminent a surgeon as the late Mr. Martineau was induced to believe the case to have been one of carotid aneurism. The preparation was at this time in the museum of Mr. Dalrymple, of Norwich.—*Royal Med. Chir. Soc. London Lancet*, July 9, 1842.

---

*Use of the Magnet in Rheumatism.*—Five cases of rheumatic affection are published in the Annals of the Medical Society of Ghent, in which, according to Dr. Beydler, the application of the magnet was very serviceable. The diseases were sciatica, rheumatic ophthalmia, and other pains which had resisted other modes of treatment, and which were diminished or removed by frictions with the magnet. One of these patients, after the cure had been effected, died suddenly, probably from rupture of an old eneurism; and another was struck with apoplexy. M. Dumont has ascertained that both poles of the magnet should not be applied at once, and narrates a case of neuralgia of the wrist, in which the north pole of the magnet removed the pain, and the south reinduced it.—*Provincial Medical Journal, June* 25, 1842.

